# Down‐regulation of SARS‐CoV‐2 neutralizing antibodies in vaccinated smokers

**DOI:** 10.1002/mco2.166

**Published:** 2022-08-12

**Authors:** Jiahui Zhang, Fei Teng, Xiaomei Zhang, Hongye Wang, Te Liang, Shubin Guo, Xiaobo Yu

**Affiliations:** ^1^ State Key Laboratory of Proteomics Beijing Proteome Research Center National Center for Protein Sciences‐Beijing (PHOENIX Center) Beijing Institute of Lifeomics Beijing China; ^2^ Emergency Medicine Clinical Research Center Beijing Chao‐Yang Hospital Capital Medical University and Beijing Key Laboratory of Cardiopulmonary Cerebral Resuscitation Beijing China

As of July 18, 2022, coronavirus disease 2019 (COVID‐19) has infected more than 562.34 million people and caused over 6.37 million deaths worldwide. Vaccination is an effective approach to help control COVID‐19 with 11.83 billion vaccine doses thus far administered.^1^ However, since the vaccines produce a heterogenous immune response, the risk of breakthrough infection is increased in vaccinated individuals who generate low levels of neutralizing antibodies (NAbs), a portion of antibodies that block the binding between SARS‐CoV‐2 and host receptor (ACE2).^2^ Therefore, it is paramount in the fight against COVID‐19 to identify the risk factors associated with the decreased immunity in individuals with vaccination, in which the smoking receives our concern due to the capability of cigarette in suppressing adaptive immune response to pathogen infection.^3^, ^4^

In order to know the influence of cigarette on the antibody responses in vaccinated individuals, we searched and manually curated the papers of our interests in PubMed database (https://pubmed.ncbi.nlm.nih.gov/) using the keywords “smoking, SARS‐CoV‐2 vaccination and antibody.” The results identified 16 papers related to our study, in which the IgM, IgG, and IgA antibodies to the SARS‐CoV‐2 Spike (S) and nucleocapsid (N) proteins were tested (Table S1). The data consistently indicated that the antibodies and NAbs in the nonsmoking group were significantly higher than that of the smoking group.^5^ Furthermore, the antibody expression was negatively correlated with the Fagerstrom test for nicotine dependence.^6^ However, the dynamic changes of NAbs in smoking and nonsmoking individuals before and after vaccination are unknown, especially to the latest Omicron variant.

In this study, we recruited 164 participants between 20 and 58 years old from Beijing Chaoyang Hospital from December 2020 to March 2021, who were divided into smoking and nonsmoking groups (Table S2 and Supporting information). The participants received two vaccine doses of an inactivated whole‐virion SARS‐CoV‐2 vaccine (Sinovac‐CoronaVac) 2 weeks apart (i.e., “Day 0″” and “Day 14″”), and their serum was collected longitudinally on days 0, 14, 42, and 90. Since the SARS‐CoV‐2 S protein is required for viral entry, serological antibodies on days 0, 14, and 42 targeting S protein and its specific domains, including subunit 1 (S1), receptor binding domain (S‐RBD), and subunit 2 extracellular domain (S2ECD), were detected using a protein array (Supporting information). Briefly, the array spotted with these S truncated proteins was incubated with serum for 30 min at room temperature. The serum antibodies bound to their target proteins on array can be visualized by a Cy3 Affinipure donkey anti‐human IgG(H+L) antibody and a microarray scanner. Prior to the data analysis, the *Z*‐score was calculated using the fluorescent signal intensity of the protein array as previously described.^7^

As shown in Figure 1, antibody levels to S protein and domains (S1, S2ECD, S‐RBD) were elevated 14 and 42 days after COVID‐19 vaccination compared to baseline in both participant groups (i.e., “Day 0″”). The expression of these antibodies was elevated faster in the nonsmoking group (*n* = 153) than the smoking (*n* = 11) group, in which the S‐RBD antibodies (*p* < 0.0001, Student's *t*‐test) showed the significant differentiation on day 42 (*p* < 0.0001, Student's *t*‐test) (Figure 1A,B, Figure S1 and S2).

**FIGURE 1:**
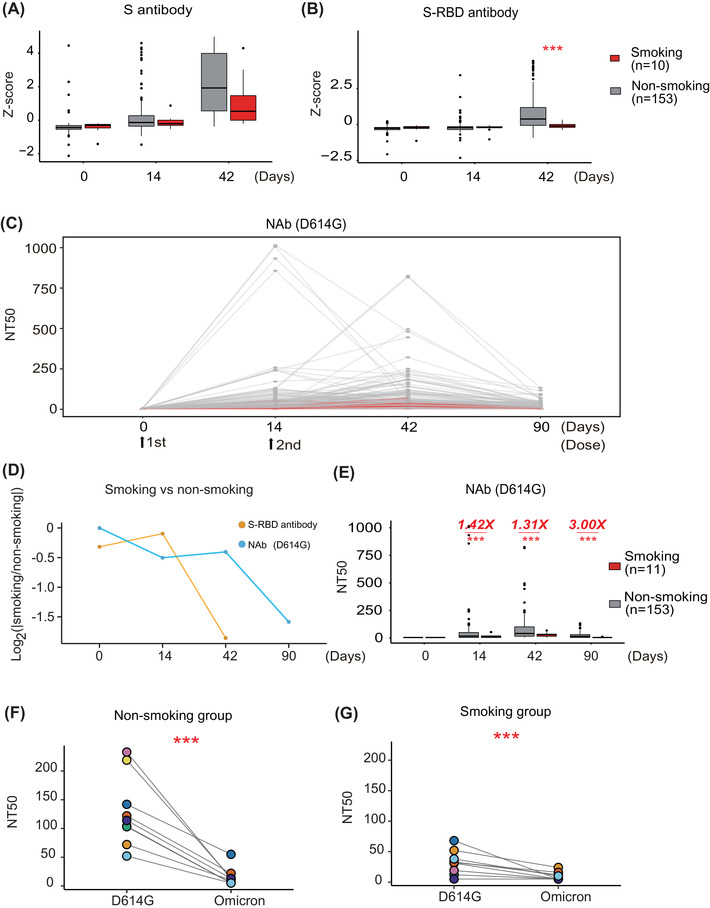
Smokers have a decreased adaptive immune response following COVID‐19 vaccination compared to nonsmokers. (A,B) Comparison of anti‐SARS‐CoV‐2 anti‐full‐length S and anti‐S‐RBD antibody expression between nonsmoking and smoking groups before and after vaccination using a protein array, respectively. *Z*‐score represents the normalized fluorescent signal of antibody binding on protein microarrays. *Z*‐score = $(x-\bar{x})/\sigma$. *x*: observed value, $\bar{x}$: mean of overall value, σ: standard deviation of overall value. (C) The longitudinal changes of neutralizing antibodies (Nabs) from 0 to 90 days in nonsmokers and smokers. (D) Relative changes of anti‐S antibodies and NAbs between nonsmoking and smoking groups before and after vaccination. (E) Comparison of SARS‐CoV‐2 NAb titers in nonsmokers and smokers before and after vaccination using a SARS‐CoV‐2 D614G pseudovirus neutralization assay. (F,G) Detection of SARS‐CoV‐2 NAb expression in nonsmokers and smokers using a SARS‐CoV‐2 Omicron pseudovirus neutralization assay, respectively. The color represents and vaccinated individuals in each nonsmoking and smoking group. NT50 represents the antibody titer that resulted in 50% pseudovirus neutralization (pNT50). *** represents the significance with a *p*‐value less than 0.01

The SARS‐CoV‐2 Spike D614G substitution is prevalent, with data showing that it increases SARS‐CoV‐2 infectivity, competitive fitness, and transmission in primary human cells.^8^ Therefore, we measured the levels of NAbs on days 0, 14, 42, and 90 following the first vaccine dose using a SARS‐CoV‐2 pseudovirus neutralization assay with the D614G substitution (Table S3 and Supporting information).^9^ NAbs continually increased after the first and second vaccine doses, peaking on day 42, and then significantly decreased until day 90 (Figure 1C).

We then compared the relative changes (ratio) of anti‐RBD antibodies and NAbs longitudinally (0, 14, 48, and 90 days) between nonsmoking and smoking groups. The results indicated that there was no significant difference between smoking and nonsmoking groups from 0 to 14 days (Figure 1D). However, the expression of anti‐RBD antibodies in the smoking group was significantly lower than that of the nonsmoking group after 14 days. Whereas the NAbs in the smoking group was significantly lower than that of the nonsmoking group after 42 days, demonstrating the specificity of cigarette to suppress NAb production through regulating humoral immunity (Figure S3). Notably, the median NAb titers in the smoking group was 1.40‐, 1.31‐, or 3.00‐fold lower than that of the nonsmoking group on days 14, 42, or 90, respectively (Figure 1E). No correlation was observed between NAbs and other factors [(i.e., age, sex, body‐mass index (BMI)] (Figure S4).

As the evolution of SARS‐CoV‐2 viral sequence, the new Omicron (B.1.1.529) and its subvariants (BA.2, BA.3, BA.4, and BA.5) continually emerged, spread and has become the predominant variant in circulation around the world. In order to know the association between smoking and NAbs to Omicron, we tested the responses of NAbs to Omicron (BA.1.) in the smoking (*n* = 8) and the nonsmoking group (*n* = 8) using pseudo virus assay and the serum samples on 42 days after second vaccination (Figure 1F, G). The results showed that the expression of NAbs was significantly decreased in both smoking and nonsmoking groups. In the smoking group, 62.5% (5/8) vaccinated individuals were below the detection limit (NT50 = 10). Comparably, 37.5% (3/8) vaccinated individuals were below the detection limit (NT50 = 10) in the nonsmoking group. The results confirm the potential influence of smoking on the NAb expression.

There have three limitations in this study. First, the number of clinical samples was limited. The results should be validated in a large different population in future. Second, the dose of cigarette in smoking participants on the suppression of immunity was not investigated due to the unavailability of clinical samples. Third, as the new variants (Omicron, BA.5.) with high infection capability continue to appear, and the relationship between the smoking and the immunity to these newly emerged SARS‐CoV‐2 variants should be investigated as well.

Altogether, our results demonstrate the capability of smoking to suppress NAb expression 14 days after inactivated vaccination, which may serve as a specific risk factor for COVID‐19 breakthrough infection following vaccination. Further investigation of smoking and how it affects NAb levels in response to COVID‐19 vaccination with a larger patient cohort and other COVID‐19 vaccines is warranted.

## CONFLICT OF INTEREST

The author declares that there is no conflict of interest that could be perceived as prejudicing the impartiality of the research reported.

## AUTHORS’ CONTRIBUTION

Xiaobo Yu and Shubin Guo designed the study. Fei Teng collected the vaccine recipients’ serum samples and clinical data. Wang Hongye, Zhang Xiaomei, and Liang Te collected the experimental data. Jiahui Zhang and Xiaobo Yu executed the statistical analysis, and visualization. Xiaobo Yu wrote the manuscript.

## ETHICS APPROVAL

This study was approved by the Institutional Review Board and Medical Ethics Committee of Beijing Chaoyang Hospital (Ethical approval number: 2020‐ke‐491). Each recipient signed an informed consent.

## Supporting information

Supporting Information

## Data Availability

All data generated or analyzed during this study are included in this published article and its supplementary information files.
